# Long noncoding RNA LINC00958 suppresses apoptosis and radiosensitivity of colorectal cancer through targeting miR-422a

**DOI:** 10.1186/s12935-021-02188-0

**Published:** 2021-09-08

**Authors:** Hong Liang, Qiuyan Zhao, Zhonglin Zhu, Chao Zhang, Hui Zhang

**Affiliations:** 1 Department of Gastrointestinal Surgery, Henan Provincial People’s Hospital, People’s Hospital of Zhengzhou University, School of Clinical Medicine, Henan University, Zhengzhou, 450003 Henan China; 2 Department of Gastroenterology, Shanghai General Hospital, Shanghai Jiao Tong University School of Medicine, Shanghai, China; 3 Shanghai Key Laboratory of Pancreatic Diseases, Shanghai General Hospital, Shanghai Jiao Tong University School of Medicine, Shanghai, China

**Keywords:** LINC00958, miR-422a, Colorectal cancer, Apoptosis, Radiosensitivity

## Abstract

**Background:**

Long noncoding RNAs (lncRNAs) have been elucidated to participate in the development and progression of various cancers. In this study, we aimed to explore the underlying functions and mechanisms of LINC00958 in colorectal cancer.

**Methods:**

LINC00958 expression in colorectal cancer tissues was examined by qRT-PCR. The correlations between LINC00958 expression and clinical characteristics and prognosis were evaluated. The biological functions of LINC00958 were detected by CCK-8, MTT, colony formation and flow cytometric analyses. RNA pulldown, RIP and luciferase reporter assays were used to confirm the regulatory effects of LINC00958 on miR-422a. Rescue experiments were performed to detect the effects of miR-422a on the roles of LINC00958.

**Results:**

LINC00958 was upregulated in colorectal cancer tissues and cell lines. High LINC00958 levels were positively associated with T stage and predicted poor prognosis. Cell experiments showed that LINC00958 promoted cell proliferation and suppressed apoptosis and sensitivity to radiotherapy in vitro and promoted tumor growth in vivo. Bioinformatics analysis predicted the binding site of miR-422a on LINC00958. Mechanistically, RNA pulldown, RIP and luciferase reporter assays demonstrated that LINC00958 specifically targeted miR-422a. In addition, we found that miR-422a suppressed MAPK1 expression by directly binding to the 3’-UTR of MAPK1, thereby inhibiting cell proliferation and enhancing cell apoptosis and radiosensitivity. Furthermore, miR-422a rescued the roles of LINC00958 in promoting MAPK1 expression and cell proliferation and decreasing cell apoptosis and radiosensitivity.

**Conclusions:**

LINC00958 promoted MAPK1 expression and cell proliferation and suppressed cell apoptosis and radiosensitivity by targeting miR-422a, which suggests that it is a potential biomarker for the prognosis and treatment of colorectal cancer.

## Introduction

Colorectal cancer is one of the most serious malignancies and the second main cause of cancer-related death worldwide [1, 2]. With advancements in early diagnosis and combined treatments, the morbidity and mortality of colorectal cancer patients aged 65 years and older have declined by 3.3% and 3.0% annually, respectively [3]. However, the incidence and death rates in individuals younger than 50 years have increased by 1% and 1.3% annually [3], respectively. To date, the potential molecular mechanisms underlying the development and progression of colorectal cancer remain ambiguous. Thus, it is urgent to explore the molecular mechanisms and identify more effective molecular targets for the early diagnosis and treatment of colorectal cancer.

Human genomes produce a great quantity of noncoding RNAs with limited protein coding potential, many of which participate in diverse biological processes. Recently, long noncoding RNAs (lncRNAs), which are larger than 200 nucleotides in length, have attracted great attention [4, 5]. Dysregulation of lncRNAs has occurred in various types of cancer. Increasing evidence has shown that lncRNAs play vital roles in the pathogenesis and progression of cancers, participating in processes such as cell proliferation, apoptosis, angiogenesis, lymphangiogenesis, cell signaling transduction and distant metastasis [6–9]. Pan et al. [10] revealed that the lncRNA FOXC2-AS1 was upregulated in colorectal cancer tissues, and si-FOXC2-AS1 suppressed cell proliferation, invasion and metastasis in vitro and in vivo. In addition, Tian et al. identified a novel lncRNA, GCMA, which was highly expressed in gastric cancer tissues and predicted poor prognosis. Additionally, they demonstrated that GCMA promoted cell proliferation, epithelial-mesenchymal transition (EMT) and cell metastasis [11]. Adjuvant radiotherapy or palliative radiotherapy contributes to downstaging or delaying tumor progression in many types of cancer [12]. Successful radiotherapy is based on a great understanding of the radiotherapy mechanisms. Recently, Wang et al. revealed that the lncRNA CCAT2 suppressed cell apoptosis and radiosensitivity in human esophageal carcinoma [13]. However, the accurate mechanisms of lncRNAs in the progression of cancers have not been elucidated. Currently, lncRNAs have been found to exert their biological functions in four different ways, namely, as signals, decoys, guides and scaffolds [14]. Wu et al. reported that the lncRNA PVT1 acted as a miRNA sponge to relieve the inhibition of VEGFA by miR-16-5p and activated the VEGFA/VEGFR/AKT pathway, thereby promoting the tumorigenesis of colorectal cancer [15]. Hua et al. [16] showed that the hypoxia-induced lncRNA AC020978 promoted cell proliferation and glycolytic metabolism in non-small cell lung cancer by directly interacting with PKM2 and enhancing the stability of the PKM2 protein. As the most important mechanism, the miRNA sponge mechanism has been a research hotspot of lncRNAs in recent years.

In this study, we found that the lncRNA LINC00958 was upregulated in colorectal cancer tissues and cell lines and significantly associated with clinicopathological features and prognosis. As the most important member of the mitogen-activated protein kinase family, MAPK1 is widely accepted to play oncogenic roles in various cancer types, such as cell proliferation, apoptosis and radiosensitivity [17–20]. We demonstrated that LINC00958 served as a miR-422a sponge and enhanced MAPK1 expression, thereby promoting cell proliferation and suppressing apoptosis and radiosensitivity. Our data suggest that the LINC00958/miR-422a/MAPK1 axis plays key roles in the cell proliferation and radiosensitivity of colorectal cancer and may be a promising candidate in the diagnosis and treatment of colorectal cancer.

## Materials and methods

### Tissues samples and cell culture

From 2013 to 2018, 63 pairs of fresh frozen samples of colorectal cancer tissues and adjacent normal tissues were collected from colorectal cancer patients in Henan Provence People’s Hospital. All the samples were snap-frozen into an RNA keeper tissue stabilizer (Vazyme Biotech Co., Jiangsu, China) and kept in a -80 °C freezer after storage at 4 °C overnight. All patients underwent radical resection without radiotherapy or chemotherapy before surgery. Written informed consent was acquired from all the subjects. The study was approved by the Institutional Review Board of Henan Provence People’s Hospital.

Human colorectal cancer cells and human normal colorectal mucosa FHC cells were purchased from the Type Culture Collection of the Chinese Academy of Science (Shanghai, China). All the cells were cultured in DMEM (HyClone, USA) with 10% fetal bovine serum (Gibco, Australia) and 1% penicillin–streptomycin in a humidified atmosphere containing 5% CO_2_ at 37 °C.

### Transfection and oligonucleotides and plasmids

To regulate the expression of LINC00958 and miR-422a, siRNA targeting LINC00958 (si-LINC00958: 5’-GTGACTAGCTTAAACTAAATT-3’) and an overexpression plasmid were synthesized; the miR-422a mimics and inhibitor were purchased from RiboBio (Guangzhou, China). When colorectal cancer cells grew to 80% confluence, they were digested with 0.25% trypsin, resuspended and seeded in 6-well plates. According to the manufacturer’s instructions, the plasmids or oligonucleotides were transfected into cells with LipofectamineTM 2000 (Invitrogen, USA) after culturing for 24 h.

### Quantitative real-time PCR (qRT-PCR)

Total RNA was extracted from cells and tissues with TRIzol (TaKaRa, Shiga, Japan) according to the manufacturer’s instructions. RNA was reverse transcribed into cDNA using the PrimeScript™ RT Master Mix Reagent Kit (TaKaRa, Shiga, Japan) for lncRNA and mRNA, and cDNA was synthesized by the PrimeScript™ RT Reagent Kit (TaKaRa, Shiga, Japan) for miRNA. Then, qRT-PCR was conducted to quantify RNA levels with SYBR Premix Ex Taq™ (TaKaRa, Shiga, Japan). GAPDH or U6 was used as an internal control. The primers were as follows: LINC00958, F: 5’-CCATTGAAGATACCACGCTGC-3’, R: 5’-GGTT GTTGCCCAGGGTAGTG-3’; MAPK1, F: 5’-CTGGACGTGCTCAGACATCG-3’, R: 5’-GGTCAGCAGGGCATC ATGTAG-3’; and GAPDH, F: 5’-CACCATTGGCAATGAGCGGTTC-3’, R: 5’-AGGTC TTTGCGGATGTCCACGT-3’. The relative expression levels of RNAs were calculated by the ΔΔCt method.

### CCK-8 and MTT assays

A total of 2000 cells were seeded into 96-well plates and cultured at 37 °C. After culturing for 0 h, 24 h, 48 h, 72 h and 96 h, the optical density of each well was measured with a CCK-8 kit or an MTT kit (Beyotime Biotechnology, Jiangsu, China) at 450 nm or 570 nm. All experiments were executed in triplicate.

### Flow cytometric analysis

Cell apoptosis assays were conducted with the Annexin V Apoptosis Detection Kit (FITC) (eBioscience, USA). Cells were treated with trypsin without EDTA. Then, the cells were washed with precooled PBS and diluted with 100 μl of binding buffer. Then, 5 μl of fluorochrome-conjugated Annexin V was added and incubated at room temperature for 15 min. After resuspension in 200 μl of binding buffer, 5 μl of PI was added. Finally, the cell apoptosis percentages were detected and analyzed by flow cytometry. All experiments were executed in triplicate.

### Radiation exposure and colony formation assay

A single dose of ionizing radiation was delivered by a Siemens 6 MV X-ray linear accelerator with a distance of 100 cm from the source skin at a dose rate of 2 Gy/min at room temperature. Two hundred transfected SW480 or HCT8 cells were seeded in six-well plates. Five dishes of cells were irradiated for 8 h with different gradient irradiation doses of 0, 2, 4, 6, and 8 Gy. After incubation for 14 days at 37 °C, the cells were fixed with 75% ethanol and stained with 0.1% crystalline purple. The number of colonies containing 50 cells or more was counted under an inverted microscope (40 × magnification, Leica, Germany). The relative survival fraction was calculated as the ratio of plating efficiency (treated) to PE (control): PE = number of colonies/number of seeded cells; SF = PE (irradiated cells)/PE (control cells). All experiments were executed in triplicate.

### Western blotting

Total protein was extracted from colorectal cancer cells using radioimmunoprecipitation assay (RIPA) buffer (Beyotime Biotechnology, Jiangsu, China) according to the manufacturer’s instructions. Protein concentrations were tested with a BCA protein assay kit (Beyotime Biotechnology, Jiangsu, China). Total protein (30 μg) was separated by SDS–polyacrylamide gel electrophoresis (SDS–PAGE) and transferred onto polyvinylidene fluoride (PVDF) membranes (Millipore, MA, USA). The membranes were blocked with 5% nonfat milk for 1.5 h at room temperature and then incubated with primary antibodies at 4 °C overnight. After incubation with secondary antibodies at room temperature for 1.5 h, protein bands were detected by ECL chemiluminescent reagent (Millipore, MA, USA). The primary antibodies were as follows: Erk1/2 (1:1000; Cell Signaling Technology), pErk (1:1000; Cell Signaling Technology), BCL2 (1:1000; Cell Signaling Technology), Bax (1:2000; Abcam) and GAPDH (1:5000; Cell Signaling Technology).

### RNA fluorescence in situ hybridization (FISH) and nuclear-cytoplasmic fractionation

RNA-FISH assays were carried out to confirm the subcellular location of LINC00958 in colorectal cancer cells. The Cy3-labeled LINC00958 probe was synthesized by GenePharma (Shanghai, China). The sequence of the Cy3-labeled LINC00958 probe was as follows: 5’-TCCTCCCATGTTTTTGTCTTCCCTACCACC-3’. Hybridization was performed according to the manufacturer’s instructions. The images were acquired using a fluorescence microscope. Nuclear and cytoplasmic RNA was isolated by a PARIS™ kit (Invitrogen, USA) according to the manufacturer’s instructions and then detected by qRT-PCR.

### RNA pull down assay

The biotinylated LINC00958 probe was produced by RiboBio (Guangzhou, China). In brief, 1 × 10^7^ SW480 cells were lysed with ultrasonication. Probe-coated beads were first incubated with the oligo probe or LINC00958 probe using C-1 magnetic beads (Life Technologies) for 2 h at 25 °C. Next, the cell lysates were incubated with C-1 magnetic beads combined with the oligo probe or LINC00958 probe (Biotin-LINC00958 of wild type: 5’-TCCTCCCATGTTTTTGTCTTCCCTACCACC-3’; Bio LINC00958 of mutation: 5’-AGGAGGGTACAAAAACAGAAGGGATGGTGG-3’) at 4 °C overnight. After washing with wash buffer three times, the RNA complexes were extracted by the RNeasy Mini Kit (QIAGEN, Germany) according to the manufacturer’s instructions. Finally, qRT-PCR was conducted to quantify the level of miR-422a.

### RNA immunoprecipitation (RIP) assay

The RIP assay was conducted in SW480 cells with the Magna RIPTM RNA-binding Protein Immunoprecipitation Kit (Millipore, Billerica, MA). SW480 cells were transfected with miR-422a mimics or negative control. After transfection for 48 h, the cells were lysed with complete RNA lysis buffer. Magnetic beads were first conjugated with human anti-AGO2 antibody or negative control mouse IgG. Then, cell lysates were rotated in RIP immunoprecipitation buffer with the above magnetic beads. The next day, immunoprecipitated RNA was incubated with Proteinase K for 30 min and extracted by TRIzol. Finally, qRT-PCR was performed to identify the level of LINC00958.

### Luciferase reporter assay

Luciferase reporter plasmids (pGL3-LINC00958 sequence, pGL3-mutant LINC00958 sequence, pGL3-MAPK1 3’-UTR sequence and pGL3-mutant MAPK1 3’-UTR sequence) were produced by HarO Life Co. (Shanghai, China). The luciferase reporter plasmids were transfected into cells with the miR-422a mimic or inhibitor. After 36 h, the activities of firefly luciferase and Renilla luciferase were detected. The relative luciferase activity was calculated as firefly luciferase activity/Renilla luciferase activity *100%.

### Animal experiments

To establish xenograft tumor models, shRNAs against LINC00958 (sh-LINC00958) and a negative control (sh-NC) were generated and cloned into lentiviruses. Then, lentiviruses were transfected into SW480 cells with 5 mg/ml polybrene for 48 h. Finally, stable SW480 cells were selected with puromycin (5 μg/ml) for 2 weeks. The knockdown efficiency was confirmed by qRT-PCR. For the in vivo tumorigenesis assay, 10 4-week-old male BALB/c athymic nude mice were randomly divided into two groups (n = 5). A total of 1.0 × 10^7^ stable SW480 cells in 150 μl of PBS were subcutaneously injected into the left inguinal region of the mice. After 10 days, tumor volumes were measured every three days until 4 weeks. Tumor volume was calculated by the following formula: tumor volume = (length × width^2^)/2. Finally, the mice were sacrificed, and the volume and weight of tumors were detected. The animal experiments were approved by the Institutional Animal Care and Use Committee of Zhengzhou University and performed according to the guidelines for the care and use of laboratory animals.

### Statistical analysis

SPSS 22.0 software was used for statistical analyses. Count data were analyzed by the χ^2^ test or Fisher’s exact test. Paired and unpaired measurement data were compared by Student’s t-test or the Mann–Whitney U test. The survival curves were calculated by the Kaplan–Meier method and analyzed by the log-rank test. A probability of 0.05 or less was considered statistically significant for all tests.

## Results

### LINC00958 is overexpressed in colorectal cancer tissues

**Fig. 1:**
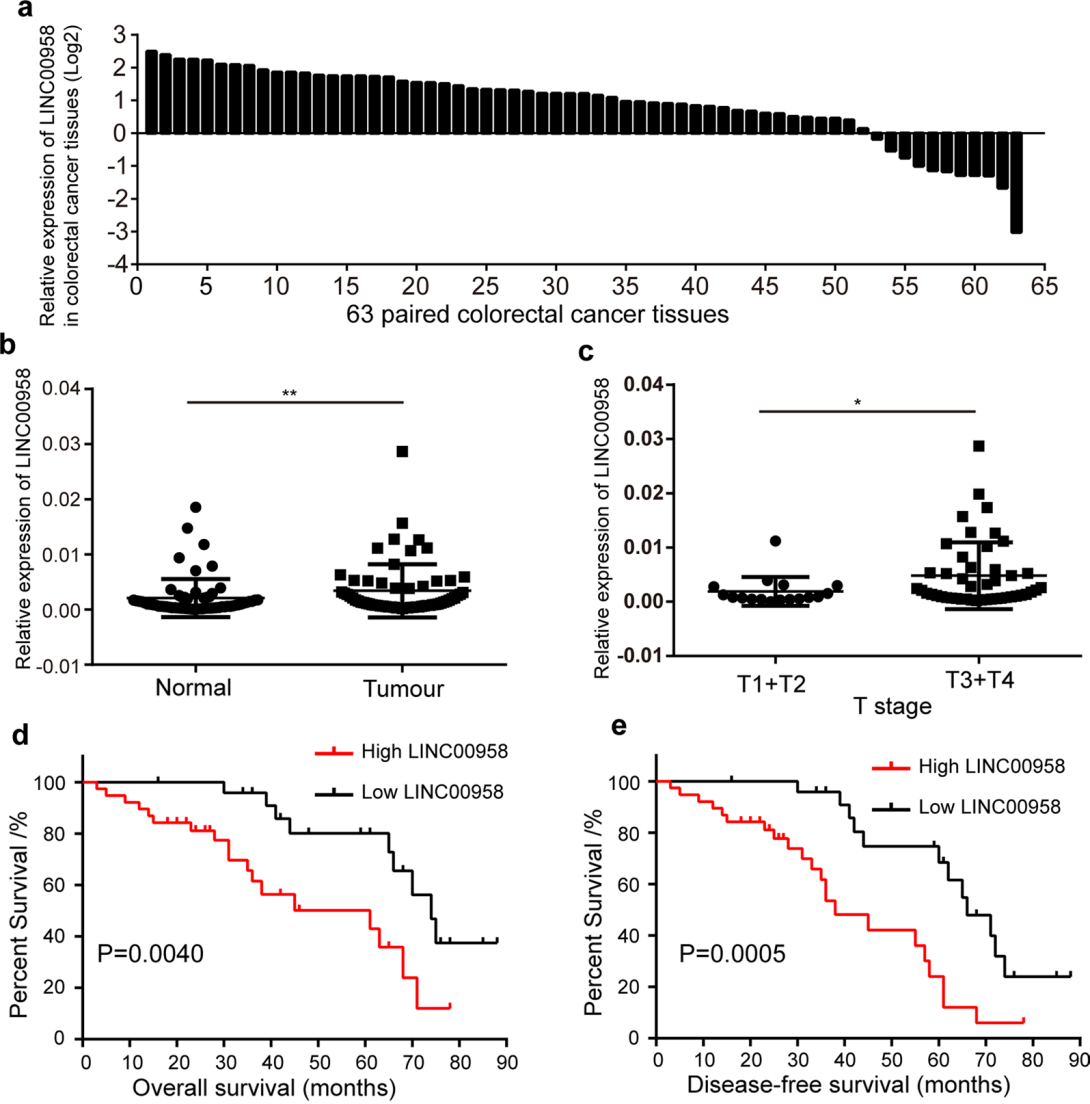
Features of LINC00958 in colorectal cancer tissues. **a** LINC00958 expression was upregulated in most (82.54%, 52/63) of colorectal cancer tissues. **b** LINC00958 expression was significantly higher in 63 colorectal cancer tissues than adjacent normal colorectal tissues. **c** The level of LINC00958 was significantly higher in colorectal cancer tissues at T3 and T4 stages than these at T1 and T2 stages. **d** and **e** Kaplan–Meier Plotter analysis revealed that high LINC00958 level predicted a poorer overall survival (**d**) and disease-free survival (**e**) for colorectal cancer patients. *p < 0.05, **p < 0.01, ^NS^p > 0.05

Previous studies showed that the level of LINC00958 was upregulated in cancers, such as hepatocellular carcinoma and oral squamous cell carcinoma, and predicted poor prognosis [21, 22]. In this study, the level of LINC00958 was detected with qRT-PCR technology in 63 paired colorectal cancer tissues and matched adjacent normal tissues. We found that LINC00958 expression was upregulated in colorectal cancer tissues compared with matched normal tissues (82.54%, 52/63) (Fig. 1a, b). Next, the correlations between the level of LINC00958 and clinicopathological features were analyzed in these 63 paired colorectal cancer tissues. The results revealed that the level of LINC00958 was positively correlated with tumor differentiation, T stage and TNM stage (Table 1, P < 0.05). The level of LINC00958 was remarkably higher for stage T3 and T4 disease than for stage T1 and T2 disease (Fig. 1c). However, there were no significant differences in colorectal cancer tissues between different differentiation and TNM stages. Kaplan–Meier survival analysis indicated that colorectal cancer patients with higher LINC00958 expression had worse overall survival (Fig. 1d) and disease-free survival (Fig. 1e) than those with lower LINC00958 expression. Overall, these results show that LINC00958 is highly expressed in colorectal cancer tissues and may act as a promising diagnostic, prognostic and therapeutic marker for colorectal cancer.

**Table 1:** Associations between LINC00958 expression and clinicopathological features in colorectal cancer (n = 63)

Parameters	Category	No.	LINC00958 expression		*χ*^*2*^	P
			High (n)	Low (n)		
Age					0.607	0.436
	< 65	34	19	15		
	≥ 65	29	19	10		
Gender					0.613	0.434
	Male	39	25	14		
	Female	24	13	11		
Differentiation					4.565	**0.003**
	Well		3	7		
	Mederate + Poor		35	18		
T stage					6.091	**0.014**
	T1 + T2		6	11		
	T3 + T4		32	14		
N stage					3.770	0.052
	N0 + N1		8	11		
	N2 + N3		30	14		
TNM stage					4.552	**0.033**
	I + II		5	9		
	III + IV		33	16		
Nerve invasion					1.724	0.189
	Yes	39	26	13		
	No	24	12	12		
Vessel invasion					0.218	0.641
	Yes	40	25	15		
	No	23	13	10		
Tumore size, cm					0.138	0.710
	< 5	27	17	10		
	≥ 5	36	21	15		

### LINC00958 promotes cell proliferation and suppresses cell apoptosis and the radiosensitization of colorectal cancer in vitro

**Fig. 2:**
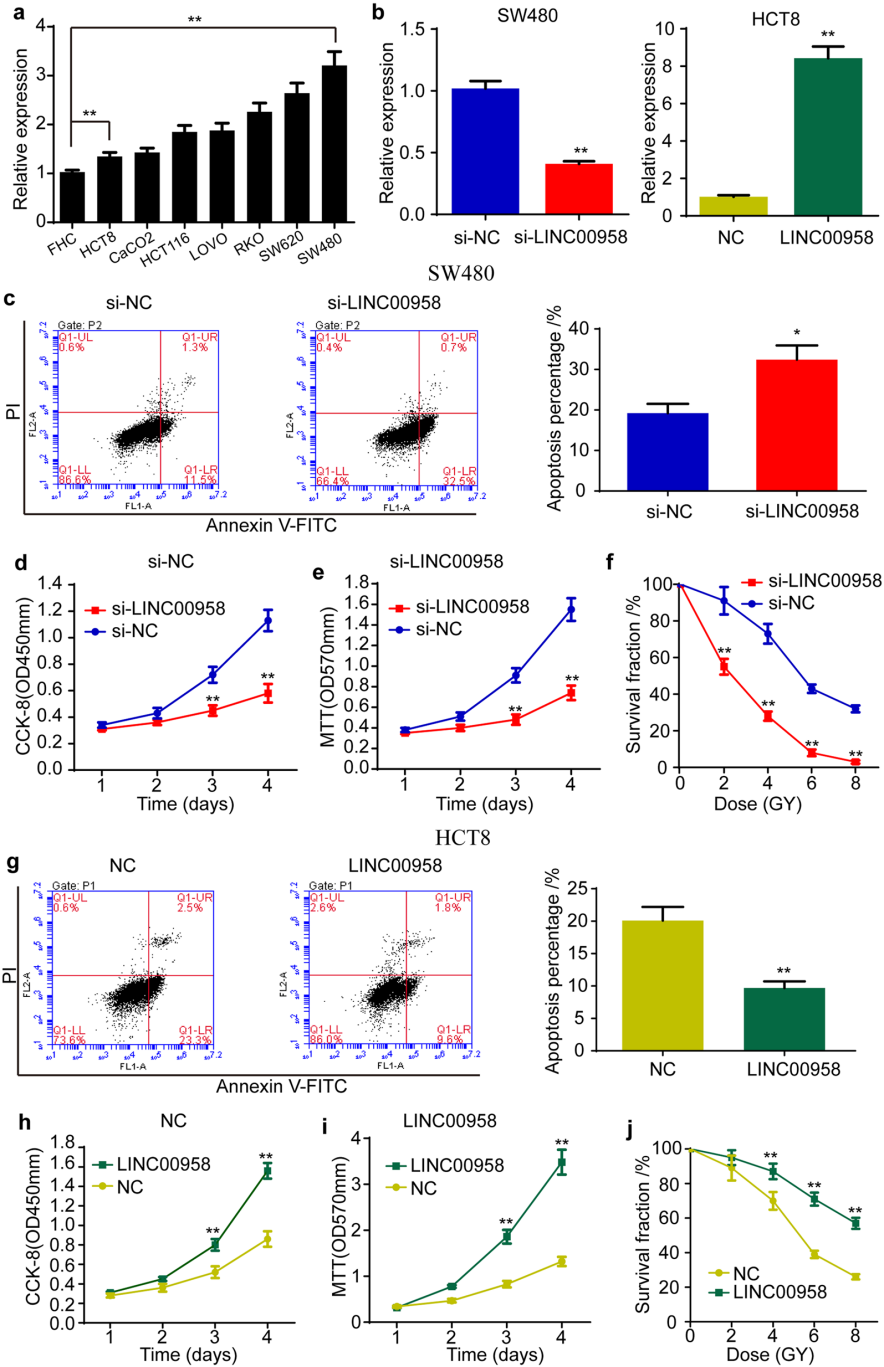
LINC00958 promotes cell proliferation, suppresses cell apoptosis and radiosensitization of colorectal cancer. **a** The expression of LINC00958 in colorectal cancer cell lines. **b** The efficiencies of siRNA targeting LINC00958 and an overexpression plasmid of LINC00958 were detected. **c** The flow cytometry assay showed that knockdown of LINC00958 significantly increased the percentage of apoptosis. **d**, **e** The CCK-8 (**d**) and MTT (**e**) results indicated that si-LINC00958 decreased the ability of cell proliferation. **f** si-LINC00958 decreased the survival fraction under different irradiation dose. **g** The flow cytometry assay showed that overexpression of LINC00958 remarkably decreased the percentage of apoptosis. **h** and **i** The CCK-8 (**h**) and MTT (**i**) results indicated that overexpression of LINC00958 increased the ability of cell proliferation. **j** Overexpression of LINC00958 increased the survival fraction under different irradiation dose. Data are reported as means ± standard deviation of three independent experiments. *p < 0.05, **p < 0.01

The expression of LINC00958 in colorectal cancer cell lines was detected using qRT-PCR. The results showed that the level of LINC00958 was higher in colorectal cancer cell lines than in FHC human normal colorectal mucosa cells (Fig. 2a). On the basis of the expression of LINC00958, SW480 and HCT8 cells were selected to further investigate the function of LINC00958. Then, siRNA targeting LINC00958 and an LINC00958 overexpression plasmid were designed and synthesized. The qRT-PCR results showed that si-LINC00958 markedly decreased the expression of LINC00958 in SW480 cells and that the overexpression plasmid significantly increased its expression in HCT8 cells (Fig. 2b). To detect the roles of LINC00958 in colorectal cancer cells, flow cytometry, CCK-8, MTT and colony formation assays were performed under different radiation doses. The results of the flow cytometry assay showed that knockdown of LINC00958 significantly increased the cell apoptosis percentage (Fig. 2c), while overexpression of LINC00958 remarkably decreased the cell apoptosis percentage (Fig. 2g). The CCK-8 and MTT results indicated that si-LINC00958 decreased cell proliferation (Fig. 2d, e), while overexpression of LINC00958 increased cell proliferation (Fig. 2h, i). In addition, we found that downregulation of LINC00958 decreased the survival fraction under different irradiation doses (Fig. 2f), and upregulation of LINC00958 increased the survival fraction under different irradiation doses (Fig. 2j), indicating that LINC00958 decreased the radiosensitization of colorectal cancer cells. Taken together, these results demonstrated that LINC00958 promoted cell proliferation and suppressed apoptosis and the radiosensitivity of colorectal cancer cells.

### LINC00958 promotes colorectal cancer cell proliferation in vivo

**Fig. 3:**
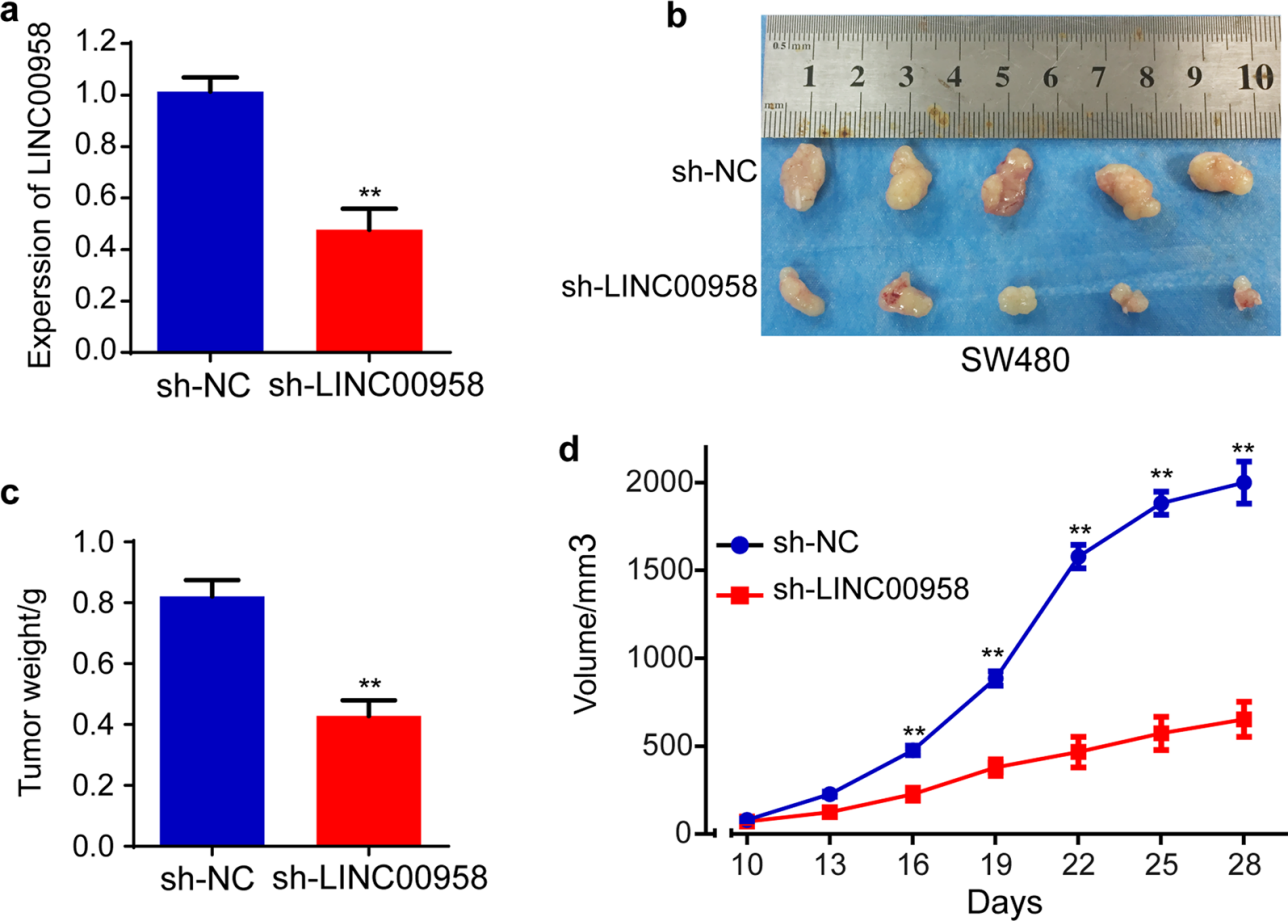
LINC00958 promotes colorectal cancer cell proliferation in vivo. **a** The knockdown efficiency of stable SW480/sh-LINC00958 cells was detected by qRT-PCR. **b** Represent pictures of tumor formation of SW480 cells. **c** The final tumor weight of SW480 cells was shown. **d** Tumor volumes of SW480 cells were measured every three days. **p < 0.01

To explore the roles of LINC00958 in colorectal cancer growth in vivo, stable SW480 cells (transfected with sh-LINC00958 or sh-NC) and a nude mouse xenograft model were constructed. The knockdown efficiency was detected by qRT-PCR. The results showed that sh-LINC00958 significantly decreased the expression of LINC00958 in SW480 cells (Fig. 3a). Representative images of SW480 cell tumor formation are shown in Fig. 3b. The weight of the SW480/sh-LINC00958 tumors was significantly lower than that of the SW480/sh-NC tumors (Fig. 3c). In addition, the volume of the SW480/sh-LINC00958 tumors was remarkably lower than that of the SW480/sh-NC tumors (Fig. 3d). Collectively, our results demonstrate that LINC00958 promotes colorectal cancer cell proliferation in vivo.

### LINC00958 acts as a miRNA sponge of miR-422a in colorectal cancer cells

**Fig. 4:**
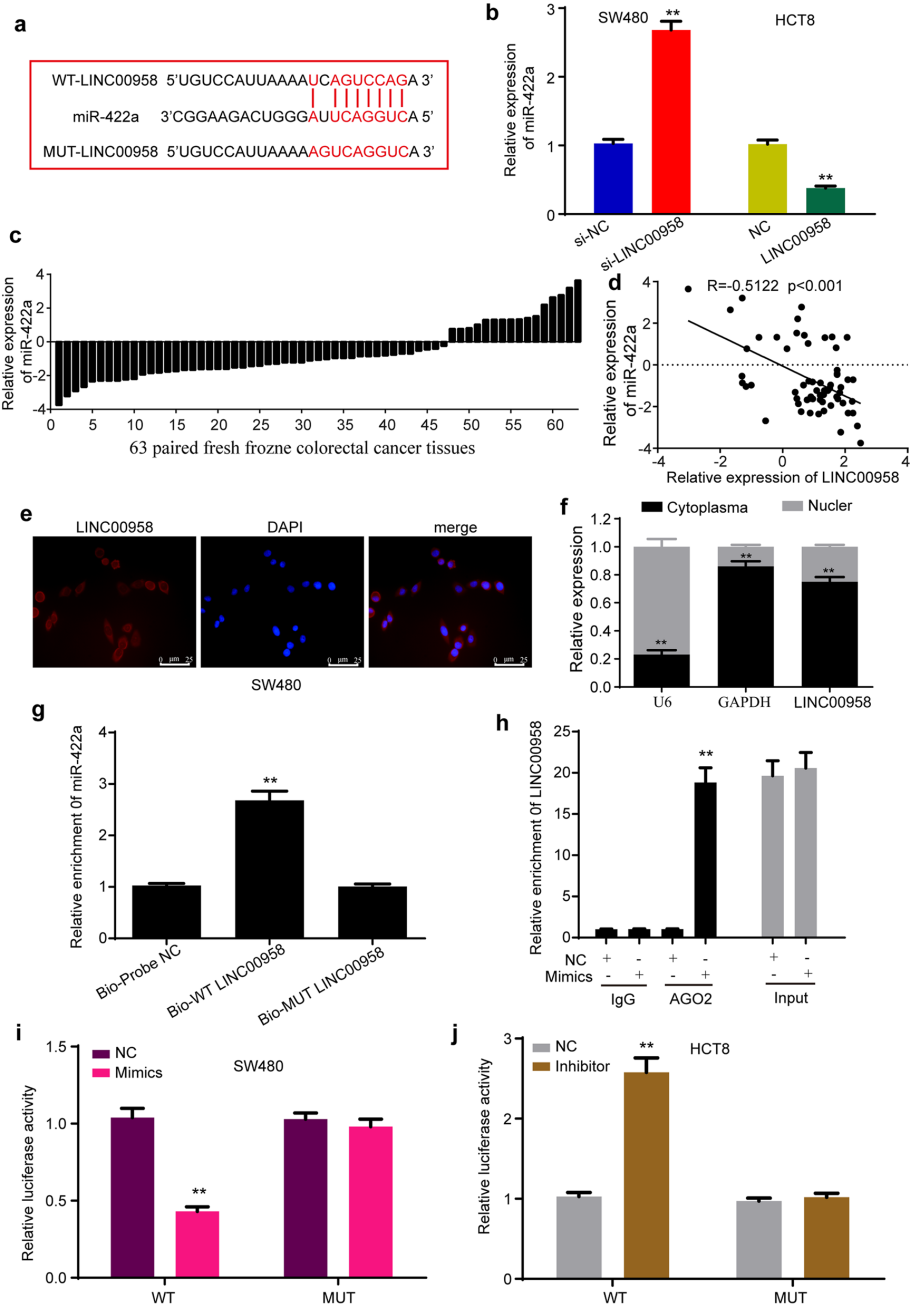
LINC00958 acts as a miRNA sponge of miR-422a. **a** Bioinformatics databases predicted that LINC00958 contained the binding site of miR-422a. **b** Expression of miR-422a after knockdown or overexpression of LINC00958 in colorectal cancer cells. **c** The expression of miR-422a in 63 paired colorectal cancer tissues. **d** Pearson’s correlation showed that miR-422a level negatively correlated with LINC00958 level in 63 paired colorectal cancer tissues (R = − 0.5122, p < 0.001). **e** RNA-FISH assays showed that most of LINC00958 was located in the cytoplasm of SW480 cells. **f** qRT-PCR results of U6, GAPDH and LINC00958 expressions in cell nucleus and cytoplasm. **g** The biotinylated RNA pull-down showed that miR-422a was pulled down by LINC00958 probe, but not by mutant LINC00958 probe, in SW480 cells. **h** RIP assay was performed with AGO2 antibody in SW480 cells transfected with miR-422a mimics or NC, and the enrichment of LINC00958 was detected. **i** miR-422a mimics significantly decreased the luciferase activity of wild type of LINC00958, but not the activity of mutation of LINC00958. **j** miR-422a inhibitor evidently increased the luciferase activity of wild type of LINC00958, but not it of mutation of LINC00958. Three independent experiments were performed for each group. All data are reported as the mean ± SD. **p < 0.01

Since lncRNAs exert biological functions mainly by acting as miRNA sponges, we explored whether LINC00958 promotes colorectal cancer progression by sponging miRNAs. To explore the potential target miRNAs of LINC00958, the TargetScan (http://www.targetscan.org) and StarBase (http://starbase.sysu.edu.cn) databases were employed to predict the underlying miRNAs and binding sites of LINC00958. Both databases showed that LINC00958 contained the binding site of miR-422a (Fig. 4a). The qRT-PCR results showed that si-LINC00958 significantly increased the expression of miR-422a in SW480 cells, and overexpression of LINC00958 decreased miR-422a expression (Fig. 4b), implying a potential correlation between LINC00958 and miR-422a. To analyze the correlation between LINC00958 and miR-422a, the level of miR-422a in colorectal cancer tissues was detected. The results showed that miR-422a expression was downregulated in most colorectal cancer tissues (74.60%, 47/63) (Fig. 4c). Further analysis indicated that the level of LINC00958 was negatively correlated with the level of miR-422a in colorectal cancer tissues (R = -0.5122, P < 0.01) (Fig. 4d). To identify the subcellular location of LINC00958 in colorectal cancer cells, RNA-FISH assays were conducted with the Cy3-labeled LINC00958 probe. The results showed that LINC00958 was mostly located in the cytoplasm of SW480 cells (Fig. 4e). In addition, nuclear-cytoplasmic fractionation assays were performed. The results showed that LINC00958 was mostly located in the cytoplasm (Fig. 4f). Considering that the location of lncRNAs impacts their functions, we speculated that LINC00958 might affect gene expression as a competitive endogenous RNA (ceRNA). Next, biotinylated RNA pulldown and RIP assays were performed to identify whether LINC00958 directly interacted with miR-422a. The RNA pulldown assay showed that miR-422a was captured by wild-type LINC00958 but not mutant LINC00958 (Fig. 4g). The RIP assay was conducted to immunoprecipitate LINC00958 with an anti-AGO2 antibody or control IgG. The results showed that LINC00958 was enriched by miR-422a mimics compared with the negative control (Fig. 4h). These data demonstrated that LINC00958 directly interacted with miR-422a. Furthermore, to investigate the regulatory effects of LINC00958 on miR-422a, we constructed two luciferase reporter plasmids with wild-type LINC00958 (WT) and mutated LINC00958 (MUT) in which the binding site of LINC00958 on miR-422a was mutated (Fig. 4a). Luciferase reporter assays showed that miR-422a mimics significantly decreased the luciferase activity of wild-type LINC00958 but not mutant LINC00958 (Fig. 4i), while the miR-422a inhibitor evidently increased the luciferase activity of wild-type LINC00958 (Fig. 4j). In general, these data demonstrated that LINC00958 acted as a miR-422a sponge by directly binding to MREs.

### MiR-422a suppresses cell proliferation and increases cell apoptosis and the radiosensitization of colorectal cancer through MAPK1

**Fig. 5:**
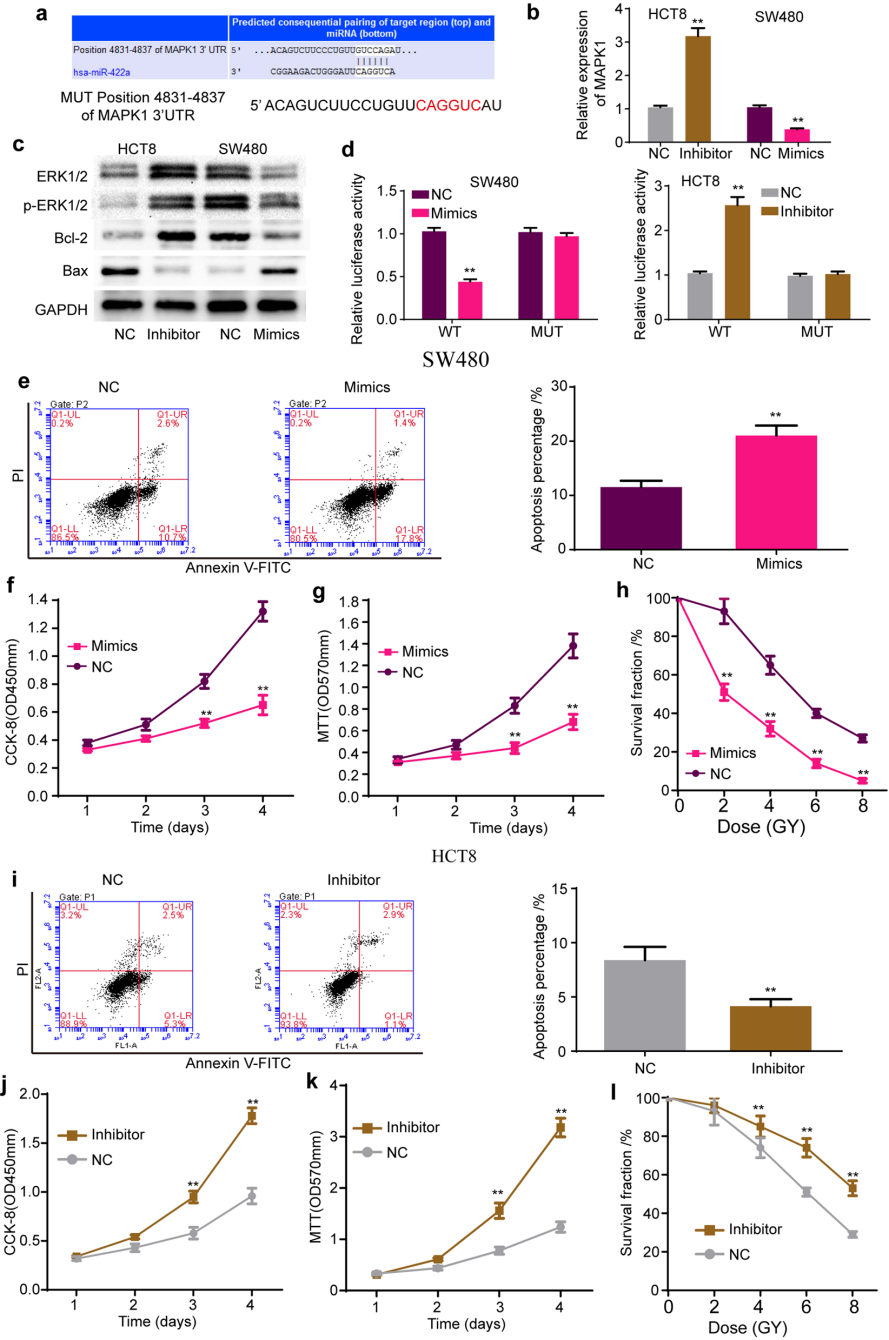
miR-422a suppresses cell proliferation, increases cell apoptosis and radiosensitization of colorectal cancer through MAPK1. **a** Bioinformatics databases predicted that MAPK1 was a target of miR-422a. **b**, **c** mRNA and protein expression of MAPK1 after knockdown or overexpression of miR-422a in colorectal cancer cells. **d** miR-422a mimics significantly decreased the luciferase activity of wild type of MAPK1, but not the activity of mutation of MAPK1. In addition, miR-422a inhibitor evidently increased the luciferase activity of wild type of MAPK1, but not it of mutation of MAPK1. **e**, **i** The flow cytometry assay showed that miR-422a mimics significantly increased the apoptosis percentage (**e**), while miR-422a inhibitor remarkably decreased the apoptosis percentage (**i**). **f**, **g**) and **j**, **k** The CCK-8 and MTT results indicated that miR-422a mimics decreased the ability of cell proliferation, and miR-422a inhibitor increased it. **h**, **l** miR-422a mimics enhanced the radiosensitization of colorectal cancer cell (**h**), while miR-422a inhibitor decreased the radiosensitization (**l**). Data are reported as means ± standard deviation of three independent experiments. **p < 0.01

To explore the potential target genes of miR-422a, the miRDB (http://mirdb.org), TargetScan and miRanda (http://www.miranda.org) databases were employed to predict underlying binding sites. All three databases predicted that the MAPK1 3’-untranslated region (3’-UTR) contained the binding site of miR-422a (Fig. 5a). Next, we explored whether MAPK1 is a target gene of miR-422a. The qRT-PCR results showed that miR-422a mimics markedly decreased the expression of MAPK1, while the miR-422a inhibitor significantly increased its expression (Fig. 5b). The Western blot analysis results indicated that overexpression of miR-422a decreased the expression of both ERK1/2 and p-ERK1/2 (MAPK1 protein), decreased the expression of Bcl-2 and increased the expression of Bax, the downstream protein of the ERK pathway, while knockdown of miR-422a increased the expression of ERK1/2, p-ERK1/2 and Bcl-2 but decreased the expression of Bax (Fig. 5c). To explore the regulatory mechanisms of miR-422a on MAPK1, luciferase reporter plasmids with wild-type MAPK1 mRNA 3’-UTR (WT) or mutant MAPK1 mRNA 3’-UTR (MUT) with mutant binding sites of MAPK1 on miR-422a were constructed (Fig. 5a). The luciferase reporter assay results implied that overexpression of miR-422a decreased the luciferase activity of WT but did not decrease that of MUT, while knockdown of miR-422a remarkably increased the luciferase activity of WT but not that of MUT (Fig. 5d). In summary, these data proved that miR-422a negatively regulated MAPK1 expression by directly binding to the MAPK1 3’-UTR.

To test the effects of miR-422a on colorectal cancer cells, flow cytometry, CCK-8, MTT and colony formation assays were performed under different radiation doses. The flow cytometry assay showed that miR-422a mimics significantly increased the apoptosis percentage (Fig. 5e), while the miR-422a inhibitor remarkably decreased the apoptosis percentage (Fig. 5i). The CCK-8 and MTT results indicated that miR-422a mimics decreased cell proliferation (Fig. 5f, g) and that the miR-422a inhibitor increased cell proliferation (Fig. 5j, k). In addition, overexpression of miR-422a enhanced the radiosensitization (Fig. 5h) of colorectal cancer cells, while downregulation of miR-422a exerted the opposite effects (Fig. 5l). In conclusion, these data confirmed that miR-422a suppressed cell proliferation and increased cell apoptosis and the radiosensitization of colorectal cancer by targeting MAPK1.

### LINC00958 promotes the progression of colorectal cancer by targeting miR-422a

**Fig. 6:**
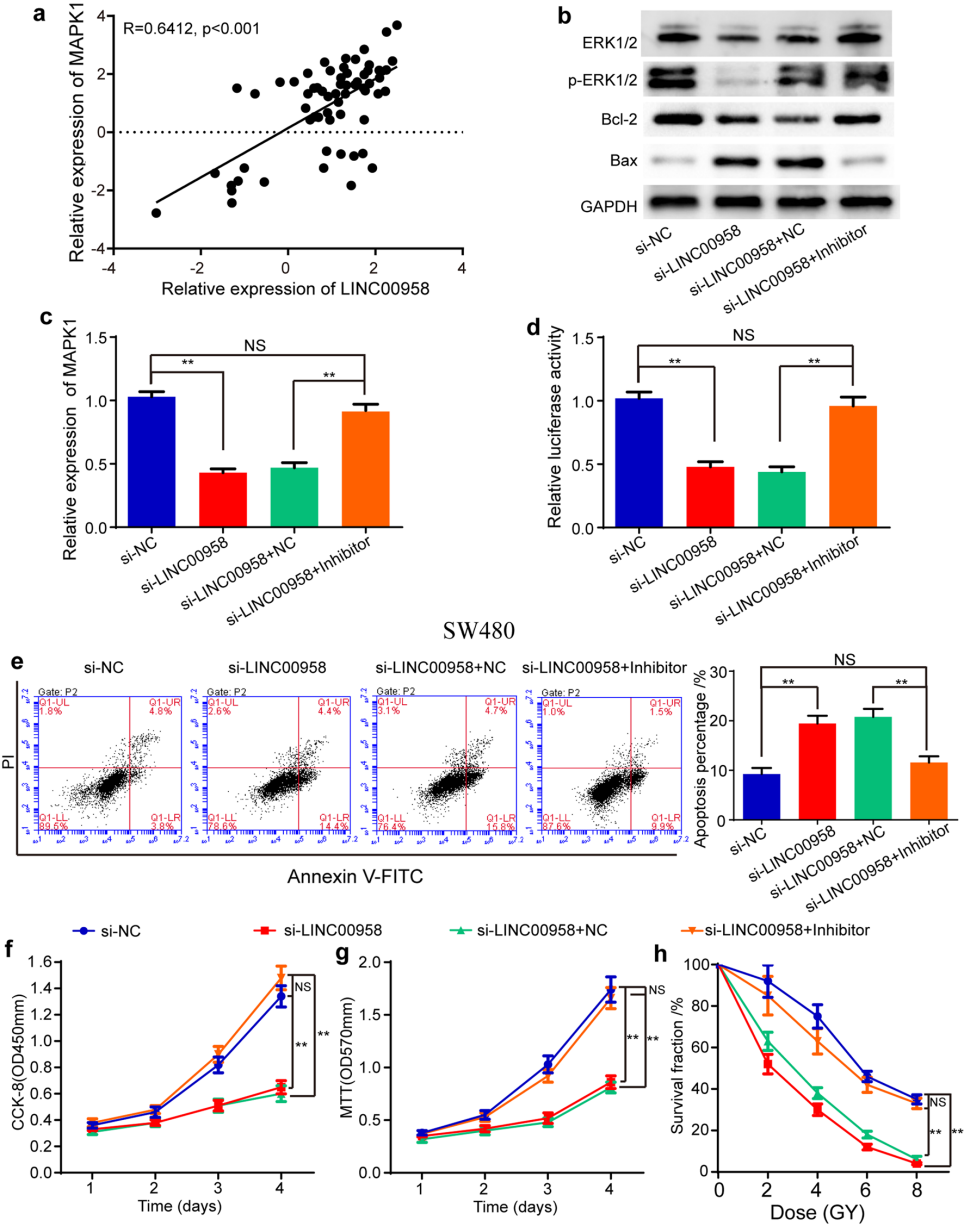
LINC00958 promotes MAPK1 expression and progression of colorectal cancer through miR-422a. **a** Pearson’s correlation showed that LINC00958 level positively correlated with MAPK1 level in 63 paired colorectal cancer tissues (R = 0.6412, p < 0.001). **b** The expression of MAPK1 protein (ERK1/2, pERK1/2), Bcl-2 and Bax after knockdown of LINC00958 and miR-422a in SW480 cells. **c** The mRNA expression of MAPK1 after knockdown of LINC00958 and miR-422a in SW480 cells. **d** The relative luciferase activity of wild type of MAPK1 mRNA 3’-UTR after knockdown of LINC00958 and miR-422a in SW480 cells. **e** The results of flow cytometry assay after knockdown of LINC00958 and miR-422a in SW480 cells. **f**, **g** The CCK-8 (**f**) and MTT (**g**) results after knockdown of LINC00958 and miR-422a in SW480 cells. **h** The survival fraction under different irradiation dose after knockdown of LINC00958 and miR-422a in SW480 cells. Data are reported as means ± standard deviation of three independent experiments. **p < 0.01

It has been revealed that lncRNAs weaken miRNA activity by competing for shared MREs, thereby regulating the expression of miRNA target genes. Therefore, we next explored whether LINC00958 could regulate the expression of MAPK1. Pearson’s correlation analysis showed that the level of LINC00958 was positively correlated with the level of MAPK1 in 63 paired colorectal cancer tissues (Fig. 6a). Knockdown of LINC00958 downregulated the expression of ERK1/2 and pERK1/2 protein and MAPK1 mRNA, downregulated the expression of Bcl-2 protein and upregulated the expression of Bax protein (Fig. 6b, c). Furthermore, si-LINC00958 decreased the luciferase activity of wild-type MAPK1 (Fig. 6d). These results implied that LINC00958 enhanced MAPK1 expression.

To test the effects of miR-422a on the roles of LINC00958 in colorectal cancer, rescue experiments were performed. The Western blot and qRT-PCR results showed that the miR-422a inhibitor relieved the suppression of si-LINC00958 on the level of ERK1/2 and pERK1/2 (MAPK1 protein) as well as the level of Bcl-2 and Bax (Fig. 6b) and MAPK1 mRNA (Fig. 6c), while the si-LINC00958 + inhibitor group was not significantly different from the si-NC group (Fig. 6b, c). Thus, the rescue experiments proved that the effects of LINC00958 on the MAPK1 pathway were purely via miR-422a. Moreover, the miR-422a inhibitor rescued the suppression of si-LINC00958 on the luciferase activity of wild-type MAPK1 (Fig. 6d). Functionally, the flow cytometry assay showed that the miR-422a inhibitor reversed the ability of si-LINC00958 to promote cell apoptosis, while the si-LINC00958 + inhibitor group was not significantly different from the si-NC group, implying that knockdown of LINC00958 increased the apoptosis percentage through miR-422a (Fig. 6e). The CCK-8 and MTT results indicated that si-LINC00958 decreased cell proliferation, while the miR-422a inhibitor relieved the ability of si-LINC00958 to inhibit cell proliferation (Fig. 6f, g). In addition, the miR-422a inhibitor increased the survival fraction under different irradiation doses downregulated by si-LINC00958, while the si-LINC00958 + inhibitor group was not significantly different from the si-NC group (Fig. 6h). Overall, these data demonstrated that si-LINC00958 suppressed cell proliferation and increased cell apoptosis and the radiosensitization of colorectal cancer by targeting miR-422a.

## Discussion

To date, many lncRNAs have been revealed to play vital roles in the development and progression of various cancers. However, the functions and mechanisms of lncRNAs in the cell proliferation, apoptosis and radiosensitization of colorectal cancer have not been clearly identified. In this study, we found that LINC00958 promoted cell proliferation and suppressed cell apoptosis and radiosensitization in colorectal cancer. Mechanistically, LINC00958 served as a ceRNA to sponge miR-422a, thereby affecting its ability to suppress the expression of MAPK1.

In recent years, noncoding RNAs have attracted researchers’ attention in the exploration of biological processes. As a class of endogenous noncoding RNAs, lncRNAs are a hotspot of gene regulation, especially in the tumor research field. Abundant lncRNAs have been successfully confirmed in various cancer cell lines and different cancer types [23–25]. Importantly, many lncRNAs have been found to participate in various biological functions, such as cell proliferation, EMT, migration, invasion and radiosensitization. Tang et al. found that the lncRNA AATBC was upregulated in nasopharyngeal carcinoma, positively correlated with poor prognosis, and promoted cell migration and invasion [26]. The lncRNA HOTAIRM1 was reported to be downregulated in papillary thyroid cancer, significantly associated with lymph node metastasis, TNM stage and better prognosis, and suppress cell growth, invasion and migration [27]. In addition, Brownmiller et al. found a significant difference in the expression of the Y chromosome lncRNA linc-SPRY3-2/3/4 between radiation-sensitive and radiation-resistant non-small cell lung cancer cells, and confirmed that knockdown of linc-SPRY3-2/3/4 promoted cell viability and resistance to apoptosis after treatment with 8 Gy [28]. In this study, we found that LINC00958 was upregulated in colorectal cancer tissues, which was in accordance with a previous study [29]. Further analysis showed that a high level of LINC00958 was positively correlated with T stage and predicted poor overall survival and disease-free survival. Functional experiments indicated that LINC00958 remarkably enhanced cell proliferation, decreased the percentage of apoptosis and the radiosensitization of colorectal cancer cells in vitro, and promoted tumor growth in vivo. As reported in a statistical analysis of cancer data, there were no significant differences in the morbidity and mortality of colorectal cancer between males and females. Therefore, both male and female mice can be used for animal experiments. Considering that male mice generally have a stronger survival ability, only male mice were used in this animal experiment. However, to confirm whether tumor growth is sex-dependent, female mice should be studied in the future. Taken together, our results demonstrated that LINC00958 promoted cell proliferation and suppressed apoptosis and the radiosensitivity of colorectal cancer. Our data suggest that LINC00958 plays an oncogenic role in the progression of colorectal cancer and may serve as a promising diagnostic, prognostic and therapeutic marker for colorectal cancer.

Accumulating evidence shows that lncRNAs are able to act as ceRNAs to sponge miRNAs, thereby protecting target genes from being suppressed or degraded [30–32]. The role of ceRNAs has been widely accepted to be the main mechanism of lncRNAs in biological processes. Wei et al. demonstrated that the lncRNA HOTAIR promoted cell growth by sponging miR-1277-5p and upregulating COL5A1 expression in gastric cancer [33]. Additionally, lncRNA-SOX2OT serves as a miRNA sponge of miR-122-5p, thereby enhancing the expression of PKM2 [34]. However, the mechanisms of LINC00958 in the progression of colorectal cancer are still unidentified. RNA-FISH and nuclear-cytoplasmic fractionation assays indicated that LINC00958 was mostly located in the cytoplasm of colorectal cancer cells, presenting its possible molecular mechanism—acting as a ceRNA, which provides a direction for further study. Furthermore, bioinformatics analysis showed that LINC00958 contained a binding site of miR-422a. Pearson’s correlation showed a negative correlation between the levels of LINC00958 and miR-422a. Next, biotinylated RNA pull-down, RIP and luciferase reporter assays confirmed that LINC00958 could directly bind to miR-422a. Further rescue experiments identified that miR-422a reversed the roles of LINC00958 in colorectal cancer progression. These data suggested that LINC00958 may exert its biological function by sponging miR-422a in colorectal cancer.

MAPK1 is the main member of the mitogen-activated protein kinase family and is an important signal transmitter from the cell surface to the nucleus. The MAPK1 pathway has been confirmed to participate in many biological processes, such as cell proliferation, apoptosis, migration and invasion [35]. Radiosensitivity is one of the main factors affecting the efficacy of radiotherapy for various cancers. Soma et al. revealed that the MAPK1 pathway was involved in the radiosensitivity of multiple tumors [36]. Bioinformatics analysis showed that the MAPK1 mRNA 3’-UTR contained the binding site of miR-422a. In our study, a series of molecular experiments demonstrated that miR-422a suppressed the expression of MAPK1 by directly binding to the 3’-UTR of MAPK1 mRNA, thereby suppressing cell proliferation and increasing cell apoptosis and radiosensitization in colorectal cancer. Additionally, we found that LINC00958 promoted the expression of MAPK1. Moreover, rescue experiments demonstrated that miR-422a reversed the regulatory effect of LINC00958 on MAPK1 expression. These data revealed that LINC00958 served as a miR-422a sponge to relieve its suppression of MAPK1 expression and enhance MAPK1 gene expression.

## Conclusion

We found that LINC00958 is upregulated in colorectal cancer tissues and positively correlated with clinicopathological features and poor prognosis. LINC00958 serves as a miR-422a sponge and enhances MAPK1 gene expression, thereby promoting cell proliferation and suppressing apoptosis and radiosensitivity. Our results provide insight into the progression and radiosensitivity of colorectal cancer and suggest that LINC00958 may serve as a promising diagnostic, prognostic and therapeutic marker for colorectal cancer.

## Data Availability

Please contact the correspondence author for the data request.
